# Nuclear Structure and Galactic γ-Ray Activity

**DOI:** 10.6028/jres.105.013

**Published:** 2000-02-01

**Authors:** Joachim Görres

**Affiliations:** University of Notre Dame, Department of Physics, Notre Dame, IN 46556 USA

**Keywords:** galactic radioactivity, radioactive lifetimes, supernovae

## Abstract

The observation of galactic γ lines following the decay of radioactive nuclei provides a direct link between nuclear physics experiments in earth-based laboratories and astrophysical observations with space-based observatories. Two examples are presented to illustrate this interplay: the measurement of the lifetime of ^44^Ti to allow an improved determination of the ^44^Ti mass of the supernova remnant Cassiopeia A from the observed γ ray activity and the measurements of excited states in ^24^Si to determine the reaction rate of ^23^Al(p, γ)^24^Si which might be important for a reduced production of ^22^Na in novae.

## 1. Introduction

Novae and supernovae are two of the main sources for galactic radioactivity. The characteristic γ radiation following the decay of some radioactive nuclei (^26^Al, ^44^Ti, ^56^Co and ^57^Co) has been observed by γ-ray detectors such as the space-based COMPTEL and OSSE instruments on board the Compton Gamma Ray Observatory (see, e.g., Ref. [1]). These observations are a direct link between nuclear physics and astrophysics. Two examples are given which describe the present experimental situation for the understanding of the formation of galactic γ-ray sources. One example is the observation of the 1157 keV γ line produced by the decay of ^44^Ti originating from the supernova remnant Cassiopeia A [1,2]. The ejected ^44^Ti mass serves as a sensitive test of the various supernova models and can be deduced from the observed γ-ray flux. However, the uncertainty of the ^44^Ti lifetime hampered a meaningful determination of the ^44^Ti mass. The previously reported lifetimes ranged from 67 years to 96 years and led to an uncertainty in the ^44^Ti mass of a factor of three. For this reason the lifetime was measured with high accuracy using a novel technique with a mixed radioactive beam. A second current problem is the discrepancy between some nova model predictions for the amount of ^22^Na produced in ONeMg-novae and recent COMPTEL observation which report upper limits that are significantly lower [3]. In novae ^22^Na is produced by the *β*-decay of ^22^Mg and a possible 2*p*-capture on ^22^Mg might lead to a significant reduction in the production of ^22^Na. To investigate this possibility more experimental information is needed to calculate the reaction rate for the 2*p*-capture more reliable. For this reason the ^28^Si(^4^He,^8^He) reaction was used to observe excited states in ^24^Si for the first time.

## 2. Lifetime of ^44^Ti

^44^Ti is created in the alpha-rich freeze-out during supernova explosions, where material cools in nuclear statistical equilibrium at low densities [4–7]. Under these conditions the build-up of heavy elements is handicapped by the slow triple-alpha process and the production of ^44^Ti depends critically on entropy and density conditions during the freeze-out [7,8]. Of special interest is therefore the observation of the 1157 keV γ-ray line from the decay of ^44^Ti. So far, the only source for this line is the supernova remnant Cassiopeia A [2]. The previously reported lifetimes for ^44^Ti range from 67 years to 96 years [9–13] and prevented a meaningful determination of the ^44^Ti mass of Cas A.

For this reason we measured the lifetime of ^44^Ti using a novel experimental approach (for details see [14]. A mixed radioactive beam of ^22^Na and ^44^Ti was implanted into a stack of Al-foils and the resulting activities were measured using a well-shielded high-resolution Ge-detector. In this method the lifetime of ^44^Ti was measured relative to the lifetime of the well known ^22^Na thus reducing the systematic uncertainties. The lifetime of ^44^Ti depends only on two ratios, the relative amount of ^44^Ti and ^22^Na in the beam, $N_{44_{\mathrm{Ti}}}/N_{22_{\mathrm{Na}}}$, and the resulting activities, $A_{44_{\mathrm{Ti}}}/A_{22_{\mathrm{Na}}}$.

A secondary radioactive ion beam was produced at the National Superconducting Cyclotron Laboratory at Michigan State University. A primary beam of ^46^Ti with an energy of *E/A* = 70.6 MeV/u was directed onto a Be target located at the target position of the A1200 projectile fragment separator[15]. The separator was operated in medium acceptance mode and optimized for maximum ^44^Ti transmission. All other *N = Z* fragments, including ^22^Na, are also transmitted to the focal plane. The experiment was run in two modes. In the first, all fragments were implanted into a stack of Al-foils which consisted of seven foils with thicknesses of 50 µm to 457 µm. ^44^Ti was implanted into the center of the third foil and ^22^Na into the center of the sixth foil. The second mode provided for particle identification of the implanted species. For this reason the primary beam intensity was reduced and a set of detectors replaced the Al-stack. The set of detectors consisted of a Si ∆*E* detector, a position sensitive Parallel Plate Avalanche Counter and a plastic detector to measure the remaining energy. This allowed the identification of the implanted particles at the implantation spot by means of energy loss, total energy and time-of-flight as well as the determination of the fragment intensities across the implantation spot.

Fragments were implanted for an accumulated time of 29 h switching every 3 h to the second mode. The mean ratio of all runs is $N_{44_{\mathrm{Ti}}}/N_{22_{\mathrm{Na}}}$ = 76.78 with 1σ-errors of ±0.73 (internal error) and ±0.78 (external error). The absolute ^44^Ti was ≈ 5×10^5^/s and a total of ≈ 5×10^10 44^Ti ions were implanted.

**Fig. 1:**
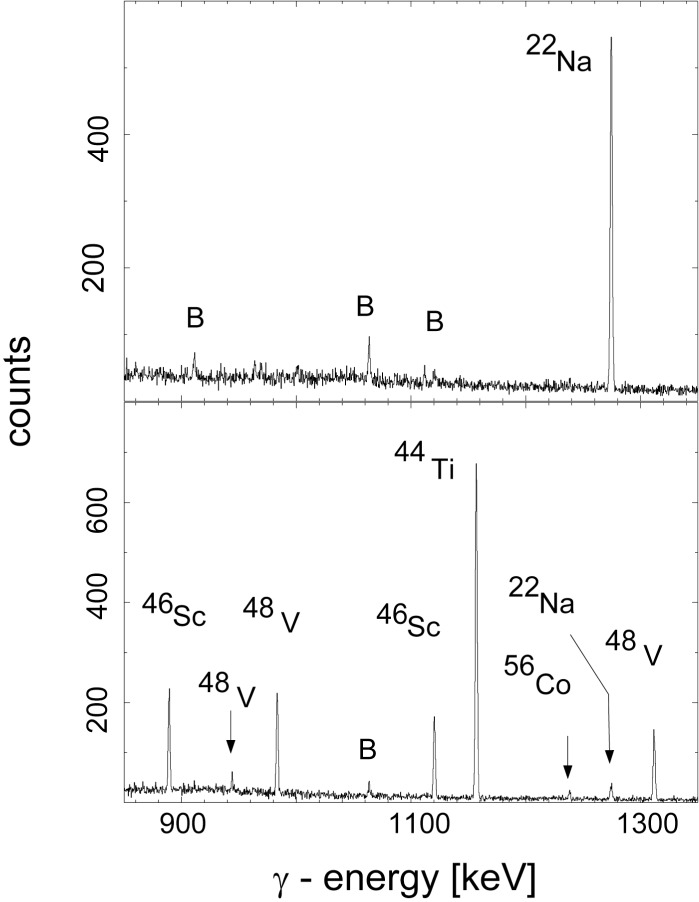
Relevant part of the γ-ray spectra with a ^22^Na foil in place (top panel) and with a ^44^Ti foil in place (bottom panel).

The specific activities of the implantation foils were measured by detecting the characteristic γ-decay lines of the radio-isotopes using a Ge detector which was completely shielded with 10 cm of Pb to reduce the room background. A sample holder allowed the placement of the foils at distances of 13.9 mm, 23.9 mm, 44.0 mm and 83.9 mm from the surface of the Gecrystal. Short-lived activities were allowed to decay during a period of three months following the implantation. The activities were measured in four cycles and during each cycle the foils were placed in each of the positions. The decay of ^22^Na and of ^44^Ti are very similar [16] and only small corrections to the ratio of the γ-intensities are needed to obtain the ratio of their activities. Figure 1 shoes the relevant part of the γ spectra with a ^22^Na foil in place (top panel) and with a ^44^Ti foil in place (bottom panel). The ratio of the ^44^Ti and ^22^Na activities were determined to $A_{44_{\mathrm{Ti}}}/A_{22_{\mathrm{Na}}}$ = 3.322±0.054. This final value includes a small correction (1 %) of the ^22^Na activity to account for secondary ^22^Na production in the implantation foils.

With these results for the ratios of the fragment intensities and the activities a ^44^Ti lifetime of $\tau_{44_{\mathrm{Ti}}}$ = (87.0±1.9) years was determined. This value is in excellent agreement with the results of several new experiments which were measured simultaneously by different groups which deduced the lifetime from the decay curve of ^44^Ti: (89.5±2.9) years [17], (85.1±0.9) years [18] and (87.6±1.7) years [19]. With the present lifetime, the observed γ flux from Cas A [2], a date of 1680 AD for the explosion and distance of 3.4 kpc [20], supernova Cas A ejected a ^44^Ti mass of (1.7±0.5)×10^–4^ *M*. The remaining uncertainty of the lifetime of ^44^Ti contributes little to the uncertainty of the ^44^Ti (6 %) which is now dominated by the experimental errors of the γ flux and the distance measurements.

## 3. Excited States in ^24^Si and ^22^Na Production in Novae

“Ne” novae are powered by explosive hydrogen burning after accretion of H-rich material onto the surface of ONeMg-white dwarfs. Material from the white dwarf, which is enriched in Ne and Mg, is mixed with the accreting hydrogen. This scenario might produce appreciable amounts of ^22^Na which can then be ejected by the nova explosion. This could lead to a γ-ray flux of the characteristic 1.27 Mev γ-ray following the decay of ^22^Na [21–25]. Recent COMPTEL observations of several close Ne novae such as Cyg 1992 and Her 1991 found only upper limits which suggest that substantially less ^22^Na is produced than predicted [3]. ^22^Na is produced by the *β*-decay of ^22^Mg which is strongly produced during hydrogen burning in the nova explosion [26]. This is mainly caused by the small proton binding energy of ^23^Al which leads to its destruction by photo-disintegration. However, a 2*p*-capture [27] on ^22^Mg could lead to destruction of ^22^Mg and thus reduce the amount ofobservable ^22^Na in novae. The strength of the 2*p*-capture depends strongly on the proton binding energies of ^23^Al and ^24^Si and the location of proton unbound, excited states in ^24^Si.

Little experimental information is available about the reactions ^22^Mg(p, γ)^23^Al and ^23^Al(p, γ)^24^Si and only a theoretical estimate about the reaction rate for the 2*p*-capture was available [28]. To obtain a more reliable reaction rate energies excited states have been measured utilizing the reaction ^28^Si(^4^He,^8^He)^24^Si (for details see [29]). The experiment was performed at the Indiana University Cyclotron Facility with an *α*-beam energy of 177.7 MeV and the reaction products were detected at the focal plane of the K600 spectrograph. Because the (^4^He,^8^He) reaction is strongly forward peaked, the spectrograph was operated in transmission mode covering an angle of 0° to 3° (3.5 msr solid angle) with the incident *α*-beam being transmitted through the spectrometer and dumped in a well shielded external Faraday cup at the focal plane.

**Fig. 2:**
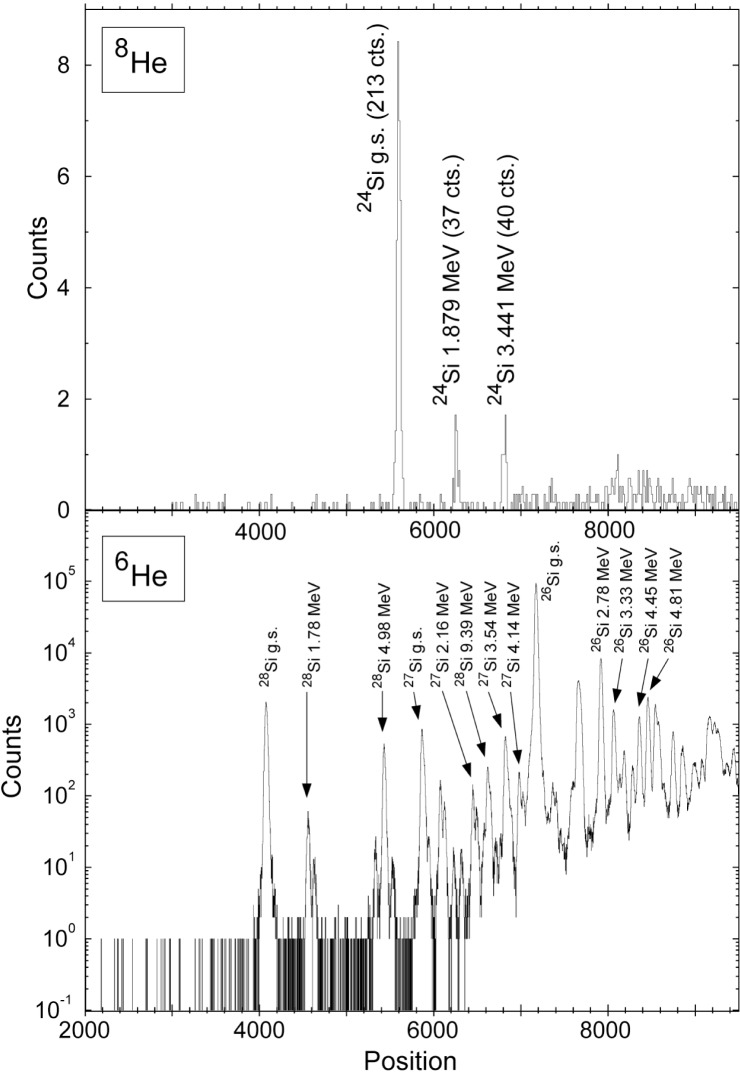
Relevant part of the ^8^He spectrum (top panel) and the simultaneously acquired ^6^He spectrum (bottom panel).

Figure 2 shows the resulting ^8^He spectrum (top panel) after an accumulated time of 70 h. The transitions to the ground state and the two first excited states are clearly visible. The spectrum displays a remarkable peak to background ratio despite the extreme forward angle and the fact that the primary beam is stopped in the focal plane. The energy calibration for the ^8^He-spectrum was obtained from the simultaneously acquired ^6^He-spectrum as well as from a separate run with a ^13^C target. The energies of the excited states relative to the ground state are (1.879±0.011) MeV and (3.441±0.010) MeV. As a consequence the resonance energy of the first resonance in ^23^Al(p,γ)^24^Si (which corresponds to the second excited state) is at (141±30) keV compared to the shell model prediction of 320 keV [28]. Despite the drastically lowered resonance energy, the resulting reaction rate is nearly the same as the previous estimate in the relevant temperature range of 0.2 GK to 0.4 GK. This is the consequence of the compensating effects of lower proton penetrabilities and the position of the resonance in relation to the position the effective energy window, the Gamow peak.

To evaluate the impact of the new reaction on the production of ^22^Na in novae, the temperature and density conditions were calculated which are necessary to process at least half the reaction flow on ^22^Mg via the 2*p*-process. These calculations indicate that the 2p-capture is too weak to cause a decrease in the ^22^Na production despite the remaining large uncertainties in the reaction rate.
